# Supplementary material for: Near-optimal P300 speller performance using large language models: A multi-model analysis with performance bounds
Source: PLoS One. 2026 Oct 1;21(10):e0349281. doi: 10.1371/journal.pone.0349281 (PMC13630319; doi:10.1371/journal.pone.0349281)
Supplement: Supplementary file 1 [file pone.0349281.s001.pdf]

S1 Appendix. Kneser–Ney smoothing formulation

Note that the convention adopted in the following terminology is that $Model('*')$ denotes the frequency of occurrence of the characters '*'. For example, *biword_model('the quick')* is the frequency of the word *'the quick'* in the biword language model. Also, as an example of the syntax of conditional probability, $p\_biword('c' \mid 'the\,qui')$ is the probability in the biword model of the character 'c' given that the partial phrase 'the qui' has been decoded.

$$p\_biword('c' \mid 'the\,qui') = \max(biword\_model('the\,quic') - d_1, 0)/biword\_model('the\,qui') + d_1 * L_1 * p\_word('c' \mid 'qui') \quad (1)$$

where L_1 is the normalizing constant equal to the number of distinct letters that follow 'the qui' in the biword model divided by the biword model count of 'the qui'. If $biword\_model('the\,qui') = 0, L_1 = 1$ to take care of out-of-vocabulary (OOV) conditions.

$$p\_word('c' \mid 'qui') = \max(word\_model('quic') - d_2, 0)/word\_model('qui') + d_2 * L_2 * p\_trigram('c' \mid 'qui') \quad (2)$$

$$p\_trigram('c' \mid 'ui') = \max(trigram\_model('uic') - d_3, 0)/trigram\_model('ui') + d_3 * L_3 * p\_bigram('c' \mid 'i') \quad (3)$$

$$p\_bigram('c' \mid 'i') = \max(bigram\_model('ic') - d_4, 0)/bigram\_model('i') + d_4 * L_4 * p\_unigram('c') \quad (4)$$

$$p\_unigram('c') = unigram\_model('c')/unigram\_model('') \quad (5)$$

where $unigram\_model('') = total\,model\,entries$ and d_1, d_2, d_3, d_4 are tunable parameters with $0 <= d_1, d_2, d_3, d_4 <= 1$. All these d values were set to the commonly used midrange default of 0.5.

Just as L_1 is a biword model normalizing constant in equation 1 whose derivation was described earlier with an example, L_2, L_3, L_4 are also normalizing constants similarly obtained from the corresponding lower-order language models. More specifically, in the example used, L_2 is the normalizing constant equal to the number of distinct letters that follow 'qui' in the word model divided by the word model count of 'qui'.

Furthermore, for satisfying the OOV conditions, if $word\_model('qui') = 0, L_2 = 1$. If $trigram\_model('ui') = 0, L_3 = 1$ and if $bigram\_model('i') = 0, L_4 = 1$ thereby accounting for the values of $L_1 \dots L_4$ in all OOV cases.

In summary, using equations 1–5, the most complex model in Fig 2 defaults to lower-level models when processing OOV characters.
